# Robot-Assisted Intracorporeal Orthotopic Ileal Neobladder: Description of the “Shell” Technique

**DOI:** 10.3390/jcm10163601

**Published:** 2021-08-16

**Authors:** Roberto Bianchi, Francesco Alessandro Mistretta, Gennaro Musi, Stefano Luzzago, Michele Morelli, Vito Lorusso, Michele Catellani, Ettore Di Trapani, Gabriele Cozzi, Matteo Ferro, Danilo Bottero, Deliu Victor Matei, Ottavio de Cobelli

**Affiliations:** 1 Department of Urology, European Institute of Oncology (IEO) IRCCS, 20141 Milan, Italy; roberto.bianchi@ieo.it (R.B.); Gennaro.Musi@ieo.it (G.M.); stefano.luzzago@ieo.it (S.L.); michele.morelli@ieo.it (M.M.); vito.lorusso@ieo.it (V.L.); michele.catellani@ieo.it (M.C.); gabriele.cozzi@ieo.it (G.C.); matteo.ferro@ieo.it (M.F.); danilo.bottero@ieo.it (D.B.); deliuvictor.matei@ieo.it (D.V.M.); ottavio.decobelli@ieo.it (O.d.C.); 2 Department of Haemato-Oncology and Oncology, University of Milan, 20122 Milan, Italy

**Keywords:** urinary bladder neoplasms, cystectomy, robotics, continent urinary reservoir, complications

## Abstract

Background: Robot-assisted radical cystectomy (RARC) with intracorporeal neobladder (ICNB) remains a very complicated, technically demanding and time-consuming surgical procedure. In the current study we describe our robot-assisted intracorporeal “Shell” neobladder reconstruction. Methods: From January 2017 to December 2019, we performed 30 intracorporeal ileal neobladder “Shell” reconstructions. We prospectively collected demographics and clinical and pathological data and retrospectively analysed perioperative, functional and oncological outcomes. Results: No conversion to open surgery or intraoperative blood transfusion was necessary. The median whole operative time was 493 min (IQR 433–530 min), ranging from 514 min (IQR 502–554 min) recorded during the first ten procedures to 470 min (IQR 442–503 min) of the last ten. The median estimated blood loss was 400 mL (IQR 350–700 mL). The median length of stay was 11 days (IQR 10–17). Both early and late complication rates were 46.7%. The high-grade early complication rate accounted for 20%, while the high-grade late complication rate was 30%. The daytime continence rate registered was 73.3%, while night-time continence rate was 60%. Conclusions: Our results demonstrated “Shell” neobladder reconstruction as a technically feasible procedure, with good functional outcomes in tertiary referral centre. Longer follow-up and larger populations are needed to validate these preliminary results.

## 1. Introduction

Radical cystectomy (RC) is the gold standard treatment for muscle invasive bladder cancer (MIBC) [1]. In the last years Robot assisted RC (RARC) rate has increased, rising from 0.6% to 32.4% of all cystectomies (2004–2017) performed in the United States [2].

Similarly, the robotic intracorporeal urinary diversion (ICUD) rate has also recently increased as a minimally invasive alternative to open surgery. However, ICUD remains a complicated, technically demanding and time-consuming surgical procedure [3]. The results reported by the International Robotic Consortium demonstrated comparable operative time, less blood loss, lower blood transfusion rate and shorter length of stay when ICUD was compared to extracorporeal urinary diversion (ECUD) [4].

Orthotopic ileal neobladder is the most used continent urinary diversion, and it is an attractive option offered to patients who are suitable for bladder reconstruction after RC. Apart from possible psychological and functional advantages, the first aim of neobladder reconstruction is to create a high-capacity reservoir, with a low endocavitary pressure, in order to minimize possible renal damage [5]. Despite the fact that several original open reconstructive techniques have been robotically replicated, unanimous consensus about the perfect robot-assisted intracorporeal neobladder (ICNB) has not been achieved yet.

In the current article, we describe our robot-assisted ICNB technique, which for its characteristic shape we named the “Shell neobladder”. In the current study, we aimed to find a simple reconstruction technique with a limited number of sutures needed. We reported the preliminary surgical, oncological and functional outcomes and complication rates of the first 30 cases performed.

## 2. Materials and Methods

### 2.1. Definition of Population and Variables for Analyses

From January 2014 to December 2019, we performed more that 100 RARCs at our institute; of these, 30 patients received the intracorporeal ortothopic ileal neobladder “Shell” reconstruction between 2017 and 2019. All procedures were performed by three highly experienced surgeons (O.dC., G.M. and D.B.). In particular, each of them had previously performed at least five hundred robot-assisted urological surgeries, one hundred open orthotopic ileal NBs and thirty RARCs. We prospectively collected data into an electronic institutional database and retrospectively analysed these data. Least squares linear regression tested for the estimated annual percentage change (EAPC) of neobladder reconstruction at RARC, relative to open RC performed in the same period.

The preoperative descriptive covariates consisted of the following: age, body mass index (BMI), clinical T-stage (Ta-Tis-T1 vs. T2) and neoadjuvant chemotherapy status (administered vs. not administered). The intraoperative covariates consisted of the following: operative time (min), estimated blood loss (EBL) and intraoperative transfusion rate. The oncologic outcomes consisted of pT-stage (T0, Ta-Tis-T1, T2, T3 or T4), pN-stage (not nodal involvement vs. nodal involvement), number of removed nodes, positive surgical margin (PSM), tumour relapse, cancer specific (CSM) and other-cause mortality (OCM). On the other hand, functional outcomes consisted of continence (defined as number of daily pads used 0–1 vs. more than 1) and potency valid for penetration (erectile dysfunction (ED), not ED or not ED with PDE5i) recovery. The postoperative covariates consisted of the following: length of stay (LOS) and catheterization days (≤14 vs. >14 days).

The main outcomes of interest consisted of overall, early (<30 days from discharge) and late complication (>30 days from discharge). Within complications, we examined overall complications and specific complication subgroups. All complications were also classified according to the Clavien–Dindo complication scale [6].

### 2.2. Description of the Surgical Technique

Robot-assisted radical cystectomy with orthotopic ileal “Shell” neobladder includes several steps and can be generally divided in two phases: demolition and reconstruction of urinary diversion. Following the description of the most important surgical steps.

Cystectomy phase: All patients are in supine position with a Trendelemburg inclination of 27 degrees. Before surgery, all pressure points are carefully padded in order to avoid vascular or nervous injuries. Before the port placement, a bladder catheter is positioned in order to drain it completely and to avoid inadvertent bladder leakage. 

Most procedures were performed with the robot da Vinci^®^ Xi (Intuitive Surgical Inc., Sunnyvale, CA, USA) due to its advanced ergonomy, although the da Vinci^®^ Si (Intuitive Surgical Inc., Sunnyvale, CA, USA) was used in some cases. A total of 6 ports were used, 3 for the robotic arms, 1 for the camera and 2 for the bedside assistant. Right robotic arm is usually equipped with a monopolar scissor, large needle driver or Tip-up grasper; the left arm is usually equipped with a bipolar robotic instrument (fenestrated bipolar, PK bipolar or Maryland bipolar device); a robotic grasper (Cadier, Prograsp) is usually used as the third robotic arm instrument. 

**Figure 1:**
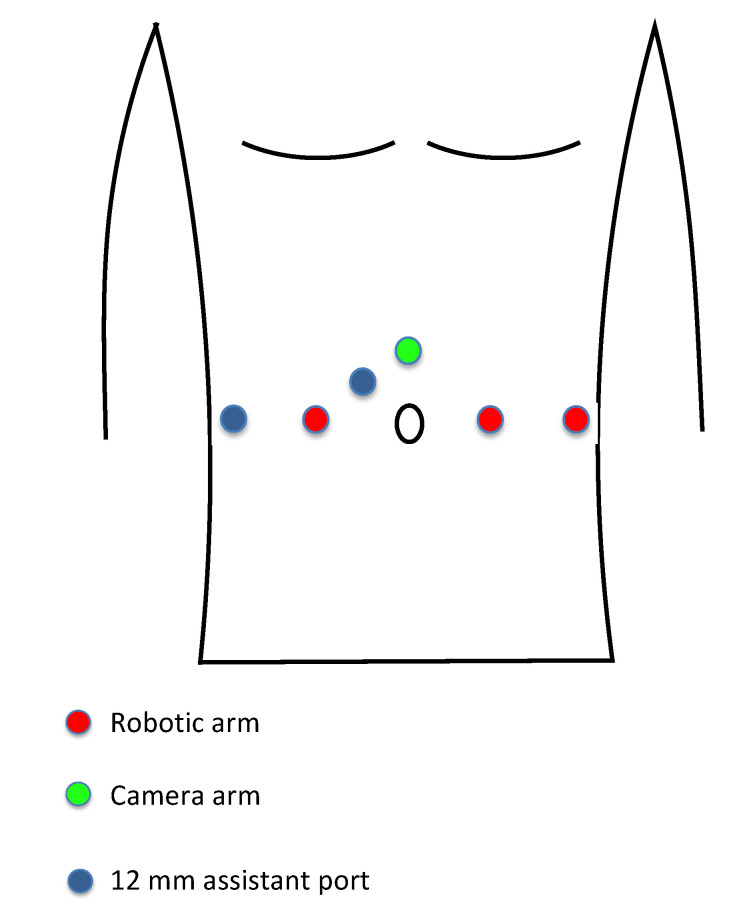
Port positioning during robotic assisted radical cystectomy.

A minilaparotomy of almost 4 cm is performed 3 cm above the umbilicus, and the Alexis^®^ wound retractor (Applied medical, Rancho Santa Margarita, CA, USA) is positioned [7]. After pneumoperitoneum induction (a 12 mmHg pressure is used for the entire procedure) the camera port is positioned through the wound retractor. All robotic arms are positioned on the same line at the level of the umbilicus. The first and second arms are positioned at 10 cm from the camera port, respectively, on the right and on the left side relative to the umbilicus. The third arm is positioned 10 cm laterally to the second arm. Two 12 mm assistant ports are positioned, one between camera port and first robotic arm and the other 10 cm laterally to the first robotic arm on the same line (Figure 1).

First, extended bilateral lymphadenectomy is performed. The lymphadenectomy boundaries are as follows: aortic bifurcation cranially, external iliac vessels laterally, internal iliac artery medially, obturator nerve and vessels caudally. Cloquet’s lymph node is usually removed. Second, ureters are freed up to the bladder wall and proximally closed with a 10 mm Hem-o-lock distally, and then they are cut by avoiding any urinary leakage. Third, when RARC is performed on male patients, the Douglas peritoneum is incised, seminal vesicles are freed and bladder vascular pedicles are ligated with Hem-o-lok and cut, or they are coagulated and cut with vessel sealer devices. An antegrade or anteroretrograde nerve-sparing procedure is then performed at the level of prostatic neurovascular bundles, avoiding any thermal or electrical neural damage. Fourth, after bladder catheter removal, the urethra is closed with a 15 mm Hem-o-lock and cut by avoiding any urinary leakage. Finally, cystectomy is completed and the bladder is removed from the wound retractor. The intraoperative pathological evaluation of urethral and ureteral surgical margins is always carried out.

**Figure 2:**
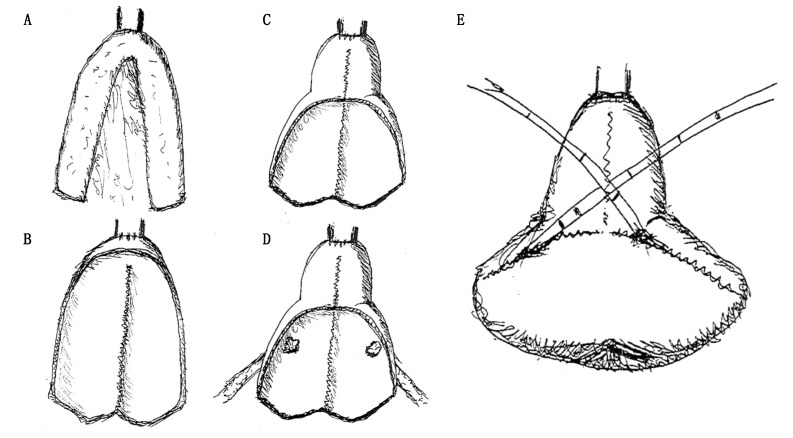
Shell neobladder reconstruction sketches. (**A**) Anastomosis between the urethra and the ileal tract identified for the future neobladder. (**B**) Configuration of the posterior plate. (**C**) Configuration of the neobladder neck. (**D**) Ureteral-neobladder anastomoses. (**E**) Configuration of the anterior plate.

Reconstruction phase: During the reconstruction phase, Trendelemburg inclination is reduced from 27 to 14 degrees. First, 40 cm of ileum was identified starting from at least 15 cm from the ileocecal valve. The ileal continuity is restored through a latero-lateral ileal anastomosis performed with surgical staplers and reinforced with running sutures or separate stitches. Second, the anastomosis between the urethra and the future neobladder is performed at 20 cm of the selected ileal tract by a bidirectional barbed suture stitch. Two running sutures, one clockwise from 6’ to 12’ and one anticlockwise, start from the posterior aspect of the anastomosis closing the left and right side, respectively (Figure 2A). Third, ileum is detubularized, and the posterior plate is configured with running barbed sutures (Figure 2B). Fourth, 10 cm of the anterior plate was sutured in order to configure a novel neobladder neck (Figure 2C). Fifth, both ureters are anastomosed at the posterior plate of the neobladder, configuring a modified split nipple with separate stitches. Successively, the ureters are bilaterally catheterized with ureteral single-J stents. These stents are finally brought outside through a suprapubic abdominal incision (Figure 2D). Lastly, the posterior plate is folded anteriorly, remembering a shell closure, and the anterior plate is configured with running barbed sutures (Figure 2E). 

Ortothopic neobladder is drained by a bladder catheter and its water-tightness is tested. On the seventh postoperative day, the first urethrocystography is performed, and the ureteral single-J stents are removed if no urinary leakage is present. At 14th postoperative day, the second urethrocystography is performed and, if negative for urinary leakage, the bladder catheter is removed.

## 3. Results

### 3.1. Preoperative and Intraoperative Characteristics of the Study Population

**Figure 3:**
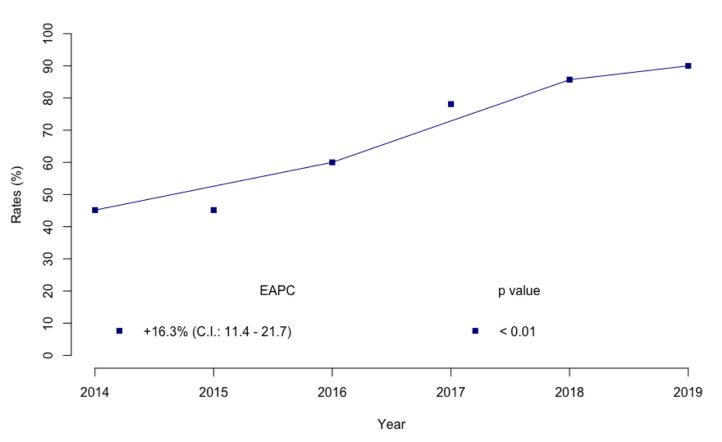
Trend of annual rate change of RARC with orthotopic ileal neobladder reconstruction.

At our Institute, the annual rates of RARC with neobladder reconstruction (45.2–85.7%; estimated annual percentage change (EAPC): +16.3%; 95% confidence interval (CI): 11.4–21.7, *p* < 0.01) sharply increased from 2014 to 2019 (Figure 3). 

The robotic intracorporeal Shell neobladder was successfully performed in all 30 male patients without any open conversion. Demographic and perioperative data are shown in Table 1. The median age of patients was 61 years old (interquartile range: 55–66); median BMI was 29.9 (IQR 24.5–29.4). Ten patients had non-muscle invasive bladder cancer and 20 patients had muscle invasive bladder cancer at transurethral resection before RC. Half of the patients received neoadjuvant chemotherapy. Overall, median operative time was 493 min (IQR 433–530 min). After stratification according to number of procedures performed (first ten procedures vs. last ten procedures), the median operative time decreased from 514 min (IQR 502–554 min) to 470 min (IQR 442–503 min). The median estimated blood loss was 400 ml (IQR 350–700), and no intraoperative blood transfusion was needed.

### 3.2. Postoperative Outcomes and Complication Rate

The median length of stay was 11 days (IQR 10–17), and 22 (73.3%) patients removed the catheter at the 14th postoperative day. Early complications (<30 days) were recorded in 14 (46.7%) patients and, out of these, 6 (20%) patients had severe complications (Clavien Dindo 3 or 4). Late complications (>30 days) were recorded in 14 (46.7%) patients and, of these, 9 (30%) patients had severe ones (Table 2). 

Specific complications were reported in Table S1 (Supplementary Materials).

### 3.3. Pathologic, Oncological and Functional Outcomes

Pathologic, oncological and functional outcomes were described in Table 3. Six (20.0%) patients were disease free after RARC, while 10 (33.3%) patients had pTa-Tis-T1, and 14 (46.7%) had pT2-T3 disease. The median number of removed lymph nodes was 26 (IQR 18–34), and two (6.7%) patients had lymph node invasion. No positive surgical margins were recorded.

After a median follow up of 15 months (IQR 12–18), tumour relapse occurred in two (6.7%) patients, while one (3.3%) patient died due to bladder cancer progression. One (3.3%) patient died due to myocardial infarction.

**Figure 4:**
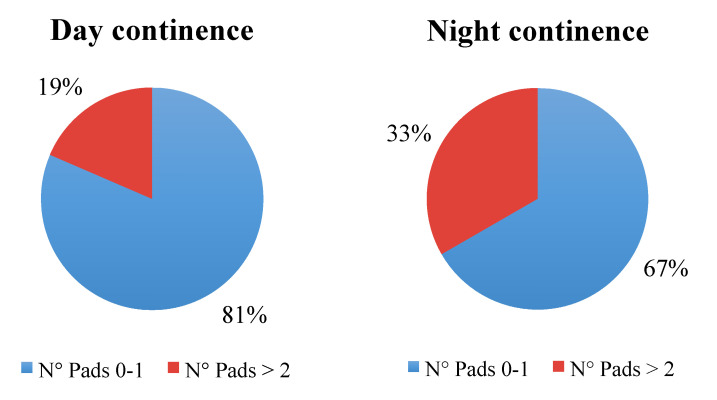
Daytime and night-time continence recovery rate after removal of patients with incomplete follow-up data.

Daytime continence was achieved in 22 (73.3%) patients, while night-time continence in 18 (60%). However, when patients with incomplete data were removed, daytime continence rate rose to 81%, and the night-time continence rate similarly rose to 67% (Figure 4). At the time of the study, no patients needed self-catheterization.

Complete potency recovery was achieved in six (20.0%) patients, while eight (26.7%) patients reported a potency recovery only after assumption of PDE5 inhibitors.

## 4. Discussion

RARC with ICNB is still considered a challenging procedure. In particular, intracorporeal reconstructive phase is limited to high volume centres, where the ICNB reconstruction technique often replicates open surgical procedure [8]. Several intracorporeal ileal reservoir variants have been described in literature [9,10,11,12,13,14,15,16]. However, the spread of intracorporeal ileal neobladder reconstruction has been slow due to the technical complexity and the increased operative time required [17]. At our institution, we have performed more than one hundred RARCs [18], and we struggled to understand the best and contemporaneously easier method to achieve a satisfactory ICNB over time. For this reason, during the last years, we studied and replicated in depth some of the previously described techniques, facing the pros and cons of each technique and pursuing the ideal robot-assisted neobladder reconstruction.

**Figure 5:**
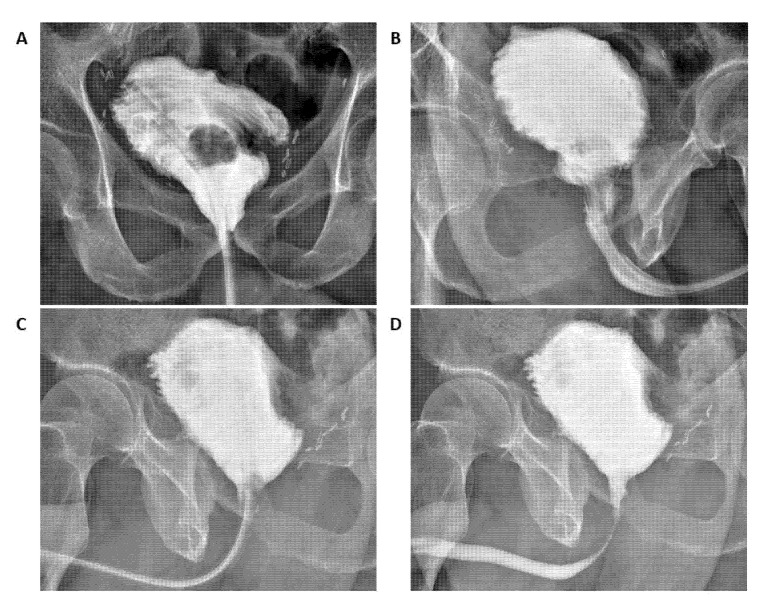
Postoperative cystogram after 14 days. Panel (**A**): coronal plane; (**B**) right to left sagittal plane; (**C**) left to right sagittal plane before catheter removal; (**D**) left to right sagittal plane after catheter removal.

In 2002 Gaboardi et al. reported a single case where laparoscopic radical cystectomy was followed by a neobladder reconstruction with both open and laparoscopic approaches [19]. During this procedure, neobladder posterior plate was obtained with a unique cranio-caudal running suture, while the closure of the anterior wall was performed with three running sutures, two latero-lateral and one cranio-caudal. The aim to reduce the number of sutures and suture directions can be encountered in other techniques previously described. “Pyramid neobladder” [14], Gaston’s “Y-neobladder” [15] and Koie’s technique [16] were models that guided our intentions, and for each of these techniques we tried to keep the pros while limiting the cons. In all of these techniques the neobladder posterior plate was synthetized with a unique cranio-caudal running suture, allowing an easier reconstruction relative to other techniques such as Studer’s technique [9] “Padua Ileal Bladder” [11] and “FloRIN” [15]. However, the first aim of neobladder reconstruction is to create a high-capacity reservoir with a low endocavitary pressure. In consequence, it is of utmost importance to achieve a spheroid shape in order to lower the endocavitary pressures [12]. This goal cannot be achieved if both the posterior and the anterior plates are reconfigured with a single direction running suture, as in the “Y-Pouch” technique [20]. Consequently, in the “Shell” technique, as well as in the “Pyramid” [12], Gaston’s “Y-neobladder” [13] and Koie’s [14] reconstructions, we reconfigured the anterior plate with two-directions running sutures in order to obtain a spheroid-like shape (Figure 5).

However, in our study we did not report urodynamic data that supported our hypothesis. Moreover, the study follow-up was not long enough to confirm this hypothesis. Nonetheless, no data showing pathological post voiding urinary residual and no need for intermittent catheterization have been recorded during the patients’ follow-up. However, a future study, such as that performed by Ferriero et al. [21] where urodynamic analyses (i.e., uroflowmetry, cystometry and urethral pressure profilometry) are performed at different follow up time points (i.e., 12 and 48 months), is mandatory to demonstrate if the Shell neobladder reaches adequate capacity and low internal pressure to protect the upper urinary tract. In several neobladder reconstructions, ureters are spatulated and directly anastomosed to the neobladder, without an antireflux technique or chimneys configurations [13]. Moreover, in the current literature, no statistically significant differences between antireflux and refluxing anastomosis have been shown [22]. In our reconstruction, ureters are anastomosed to the neobladder posterior wall forming a modified split nipple with separate stitches. Furthermore, no antireflux ureteroileal anastomosis and/or chimneys are performed.

Radical cystectomy is a procedure that possesses high morbidity regardless of surgical approach, and orthotopic neobladder is associated with higher risk perioperative complication [23]. The risk of complication for open radical cystectomy could be more than 60% and high-grade complication up to 40% [24]. Our overall complication rate was 46.7% with 30% of high-grade complications. The rate of complications reported in the current study is consistent with the recommendation of Pasadena Consensus Panel, which aims at a high-grade complication rate of 30% or lower after 100 cases per surgeon [25]. 

For those patients whose functional data could be retrieved, daytime continence rate was 81% and night-time continence rate was 67% at 12 months of follow-up. It is difficult to compare functional results with those already existing in literature, and it is difficult to draw definitive conclusions. Functional outcomes might not only be influenced by patient characteristics, such as existing comorbidities and prior treatments, but also by surgeon experience, hospital volume and technique adopted. Moreover, the difference in the definition of continence recovery used in previous studies is the major culprit of this variability. Actually, the most used definition of daytime and night-time continence is the number of pads needed (0 to 1 safety pad). Continence rate recorded in the current study is comparable to those of previous studies that focused on neobladder reconstruction at RARC. In these studies, continence rates for RARC with orthotopic neobladder reconstruction ranged from 73.3% to 100% during the daytime and 51.4% to 73.0% at night-time [11,13,24,26,27,28].

Despite the novelty and strengths of our reports, important limitations need to be acknowledged. First, our data represent a retrospective analysis with high potential for selection biases. In particular, surgical characteristics, such as difference in reconstruction shape and learning curve, could impair intraoperative and postoperative outcomes. However, all surgeons involved were highly experienced, thus limiting surgical residual biases. Furthermore, biases from unassessed covariates in patient selection, such as preoperative functional and socioeconomic status, as well as social support or lack of intraoperative data, such as time required for neobladder reconstruction, may have affected the quality of the results. Last, we did not perform urodynamic evaluation of the neobladder. However, we did not record pathological post voiding urinary residual or the need for self-catheterization. Regarding complication rate, although accessible outside records were reviewed, it is possible that some complications were missed from patients who were lost at follow-up. Finally, quality-of-life assessments were not included due to lack of pre-intervention data from validated surveys. In consequence, our findings require prospective validation in future randomized trials. 

## 5. Conclusions

Our results showed “Shell” neobladder reconstruction as a technically feasible procedure, with good functional outcomes in a tertiary referral centre. Longer follow-up and larger populations are needed to validate these preliminary results.

## Figures and Tables

**Table 1:** Preoperative and intraoperative outcomes of 30 patients with non-metastatic bladder cancer treated with robot-assisted radical cystectomy and Shell-shape ileal ortothopic neobladder reconstruction.

		Overall (*n* = 30)
Age (years)	Median	61
	IQR	55–66
Body mass index (kg/m^2^)	Median	26.9
	IQR	24.5–29.4
T-stage	Ta-Tis-T1	10 (33.3%)
	T2	20 (66.7%)
Neoadjuvant CHT	Administered	15 (50.0%)
	Not administered	15 (50.0%)
Operative time (min)	Median	493
	IQR	433–530
Estimated blood loss (mL)	Median	400
	IQR	350–700

Interquartile Range (IQR).

**Table 2:** Postoperative outcomes of 30 patients with non-metastatic bladder cancer treated with robot-assisted radical cystectomy and Shell-shape ileal ortothopic neobladder reconstruction.

		Overall (*n* = 30)
Length of stay (days)	Median	11
	IQR	10–17
Catheterization days	≤14 days	8 (26.7%)
	>14 days	22 (73.3%)
Complication	Not occurred	22 (73.3%)
	Occurred	8 (26.7%)
Early complication (<30 days from discharge)	No complication	16 (53.3%)
	Clavien-Dindo 2	8 (26.7%)
	Clavien-Dindo 3a	3 (10.0%)
	Clavien-Dindo 3b	2 (6.7%)
	Clavien-Dindo 4a	1 (3.3%)
Late complication (>30 days from discharge)	No complication	16 (53.3%)
	Clavien-Dindo 1	1 (3.3%)
	Clavien-Dindo 2	4 (13.4%)
	Clavien-Dindo 3a	5 (16.7%)
	Clavien-Dindo 3b	3 (10.0%)
	Clavien-Dindo 4a	1 (3.3%)

**Table 3:** Oncologic and functional outcomes of 30 patients with non-metastatic bladder cancer treated with robot-assisted radical cystectomy and Shell-shape ileal ortothopic neobladder reconstruction.

		Overall (*n* = 30)
pT-stage	T0	6 (20.0%)
	Ta-Tis-T1	10 (33.3%)
	T2	8 (26.7%)
	T3	6 (20.0%)
pN-stage	Absence of lymph node invasion	28 (93.3%)
	Lymph node invasion	2 (6.7%)
Number of removed lymph nodes	Median	26
	IQR	18–34
Tumour relapse	Yes	2 (6.7%)
	No	28 (93.3%)
Cancer specific mortality	Yes	1 (3.3%)
	No	29 (96.7%)
Other cause mortality	Yes	1 (3.3%)
	No	29 (96.7%)
Daytime continence	0–1 pad	22 (73.3%)
	≥2 pads	5 (16.7%)
		3 (10.0%)
Night-time continence	0–1 pad	18 (60.0%)
	≥2 pads	9 (30.0%)
	Not assessable	3 (10.0%)
Potency recovery	Erectile dysfunction	10 (33.3%)
	Potency with PDE5i	8 (26.7%)
	Complete potency recovery	6 (20.0%)
		6 (20.0%)

## Supplementary Materials

The following are available online at https://www.mdpi.com/article/10.3390/jcm10163601/s1, Table S1: Postoperative specific complications of 30 patients with non-metastatic bladder cancer treated with robot-assisted radical cystectomy and orthotopic ileal Shell neobladder reconstruction.
