# Supplementary material for: Robot-Assisted Intracorporeal Orthotopic Ileal Neobladder: Description of the “Shell” Technique
Source: J Clin Med. 2021 Aug 16;10(16):3601. doi: 10.3390/jcm10163601 (PMC8397133; doi:10.3390/jcm10163601)
Supplement: Supplementary file 1 [file jcm-10-03601-s001.zip › jcm-1286258-supplementary.pdf]

Table S1. Postoperative specific complications of 30 patients with non-metastatic bladder cancer treated with robot-assisted radical cystectomy and orthotopic ileal Shell neobladder reconstruction.

**Type of Event**	***n* (%)**
Early event (< 30 day from discharge)	
Fever	1 (3.3%)
Transfusion	3 (10%)
DVT or PE	2 (6.7%)
Atrial fibrillation	2 (6.7%)
Lymphocele	1 (3.3%)
Ureteric anastomotic leak	2 (6.7%)
Urethral anastomotic leak	1 (3.3%)
Hydronephrosis or ureteral stricture	1 (3.3%)
Intestinal anastomotic leak or perforation	1 (3.3%)
Intestinal obstruction	2 (6.7%)
Late event (> 30 day from discharge)	
Electrolyte disorder	1 (3.3%)
Infections	4 (13.3%)
Cardiovascular event	1 (1.7%)
Hydronephrosis or ureteral stricture	5 (16.7%)
Lymphocele	1 (3.3%)
Hematuria	1 (3.3%)
Intestinal anastomotic leak or perforation	1 (3.3%)

DVT: deep venous thrombus; PE: pulmonary embolism.
